# The Roles of Perceived Physical Education Competence, Enjoyment, and Persistence on Middle School Students’ Physical Activity Engagement

**DOI:** 10.1177/00315125231178341

**Published:** 2023-05-24

**Authors:** Jianmin Guan, Ping Xiang, William M. Land, Xiaofen D. Hamilton

**Affiliations:** 1 Department of Kinesiology, 12346 University of Texas at San Antonio, San Antonio, TX, USA; 2 Department of Kinesiology, 14736 Texas A&M University, College Station, TX, USA; 3 Department of Curriculum and Instruction, 12330 University of Texas, Asutin, TX, USA

**Keywords:** motivation, gender, grade, physical activity, structural equation model

## Abstract

We examined gender and grade differences in the relationship between students’ perceived competence, their enjoyment of physical education (PE), and their PA persistence on the frequency of their physical activity (PA). We also used structural equation modeling to assess the direct, indirect, and total effects of perceived competence and PE enjoyment on PA frequency through the mediator of PA persistence. Participants were 223 middle school students (115 boys, 108 girls) in grades 7 and 8. We found that, regardless of grade level, girls had lower perceived competence and PE enjoyment than boys. Both perceived competence and PE enjoyment had significant direct and positive connections to persistence, but they had no significant indirect effects on PA frequency through the mediator of persistence. These findings highlight the need for physical educators to be aware of gender differences in perceived competence and PE enjoyment, and the important roles these factors have in enhancing students’ PA participation.

## Introduction

The positive impact of physical activity (PA) on students’ physical, psychological, and social well-being have been well documented (Janssen & LeBlanc, 2010; Poitras et al., 2016). According to the U.S. Centers for Disease Control and Prevention (CDC, 2022), regular PA not only reduces the risk of disease, but also improves everyday functioning. More importantly, adolescents who are physically active tend to continue active lifestyles through adulthood (Sallis & McKenzie, 1991; Shepherd & Trudeau, 2000). Despite these positive benefits, however, many adolescents have a sedentary lifestyle, and their interest and participation in PA has tended to decline, particularly in the context of the COVID-19 pandemic (for a review, see Gao & Lee, 2022). To improve adolescents’ engagement in PA through positive physical education (PE) experiences, researchers must identify the motivational determinants of students’ PA behaviors.

Among the motivational determinants documented in the research literature (Eberline et al., 2018; Fairclough, 2003; Gao, 2008; Ghorbani et al., 2020), perceived competence and enjoyment have emerged as key motivational determinants of students' engagement in PA, and are considered the most consistent modifiable correlates of PA for children or adolescents (Sallis et al., 2000). Moreover, interventions targeting enjoyment may be most effective in promoting students' participation in PA (Sallis & Owen, 1999). According to Self-determination Theory (Deci & Ryan, 2000), people have three basic psychological needs: autonomy, competence, and connection/relatedness that underlie one’s growth and development, and that once satisfied, can motivate people to make more effort in their assigned tasks. Perceived competence is one of these three basic psychological needs; it can be conceptualized as an individual’s belief in their own ability in various achievement domains (Horn, 2004). Individuals with higher perceived competence in PE are more likely to participate in PA at school (Fairclough, 2003; Gao, 2008; Shen et al., 2018) and during leisure time outside of school (Carroll & Loumidis, 2001; Eberline et al., 2018). Generally, individuals with higher perceived competence make more effort to participate in PA, while individuals with lower perceived competence may invite ridicule from peers and exclusion from team activities. Consequently, these negative experiences influence young people’s affective reactions to PA and can lead them to withdraw from PA participation.

Gender differences in perceived PE competence have been consistently reported. Ghorbani et al. (2020), for instance, studied gender differences in the relationship between perceived competence and PA among Iranian middle school students, and found that boys reported higher perceived competence and more PA both inside and outside of school settings than girls. Similar findings were also reported for elementary school students (Carroll & Loumidis, 2001). Moreover, gender differences in perceived competence were associated with participants’ age in PE settings. Van Wersch et al. (1990), for example, examined how physical competence was perceived by 11–18 year old boys and girls; and they discovered a significant gender difference across all age categories. Specifically, their research demonstrated that boys' perceived competence remained consistent over time, whereas girls' perceived competence decreased as they got older, especially after the age of 14.

In addition to perceived competence, PE enjoyment has been found to be an important variable for promoting young people’s PA participation (CDC, 1997). PE enjoyment has been used as a predictor of PA involvement (for a review, see Sallis et al., 2000), with the level of PE enjoyment a factor in a student’s inclination to join an individual or team activity (Brazendale et al., 2015). For example, Brazendale and colleages (2015) found that children in grades 5 through 8 who had low PE enjoyment spent less out-of-school leisure time in team PA participation, but spent more time in individual activities than did those with high PE enjoyment. Additionally, gender differences in PE enjoyment have also been observed, with girls reporting lower interest and enjoyment in PE than boys (Brazendale et al., 2015; Carroll & Loumidis, 2001; Ghorbani et al., 2020). Moreover, this gap has been shown to grow with age. Cairney et al. (2012), for example, explored the relationship between gender and PE enjoyment in a sample of school-aged children across grades 4 to 6. Their study revealed that PE enjoyment declined over time among girls, but remained constant in boys over that same period.

Considerable research has shown a close relationship between perceived PE competence and enjoyment. Regardless of gender, perceived competence is positively related to enjoyment and a feeling of satisfaction with PA involvement (Eberline et al., 2018; Fairclough, 2003). Students with higher perceived competence are more likely to show increased enjoyment and effort in PA. In contrast, students who view themselves as lacking skills rarely have enjoyable PE or PA experiences during participation. The positive relationship between perceived competence and enjoyment indicates that these two variables can work together in increasing students’ PA adherence and positive attitudes toward PA. However, past studies that examined the relationship between perceived competence and enjoyment in PE with such outcome variables as effort (e.g., Williams & Gill, 1995), academic persistence (e.g., Brubacher & Silinda, 2019), and PA engagement (e.g., Ghorbani et al., 2020) focused on the mediating roles of these variables. Few studies have considered perceived competence and enjoyment as exogenous variables in a structural model when examining the relationship between them and various outcome variables. Given their strong link to PA in adolescents, further investigation of perceived competence and enjoyment in PE is warranted.

In addition to determinants of PA, researchers must examine potential mediators of these relationships. By examining the effect of mediator variables on the relations between exogenous variables and endogenous variables, investigators may better understand the antecedent conditions that influence adolescents’ PA behaviors. Such an understanding would help when implementing an effective PE program to improve out-of-school PA involvement. In this study, we selected persistence as a mediator to examine its role and impact on PA. Persistence refers to a continued investment in the process of learning in the face of difficulty. Previous researchers have examined persistence as an outcome variable of achievement goals in motivational research in PE settings (Guan et al., 2006, 2020). Additionally, many investigators have examined the combined impact of perceived competence and enjoyment on academic persistence (Brubacher & Silinda, 2019; Hardre & Reeve, 2003; Lavigne et al., 2007). These findings revealed that perceived competence and enjoyment were two significant positive predictors of academic persistence. Few studies, however, examined persistence as an endogenous variable of perceived competence and enjoyment in PE. Additionally, few studies have investigated the mediating role of persistence between perceived competence or enjoyment and PA engagement.

Previous research has significantly enhanced our understanding of gender and age differences in perceived competence and PE enjoyment, and their impact on adolescents’ PA engagement. However, most published studies have targeted student populations outside the United States, and this is particularly true of middle school students. To our knowledge, few prior investigators have applied a structural path analysis model to explore the relationships between U.S. students’ perceived PE competence, enjoyment, persistence, and leisure time PA engagement. More specifically, few studies have explored the direct and indirect effects of perceived competence and enjoyment in PE on students’ leisure time PA participation through persistence as a mediator variable. Therefore, the primary purpose of this study was to examine gender and grade level differences in middle school students’ perceived competence, enjoyment, persistence, and leisure time PA participation. Based on previous research, we hypothesized that (a) boys would report higher perceived competence and enjoyment in PE, (b) both perceived competence and enjoyment in PE would decline over time among girls but remain stable in boys, and (c) students with high competence and enjoyment in PE would be more likely to engage in leisure time PA. A secondary purpose of our study was to employ a structural path analysis model to assess the direct, indirect, and total effects of perceived competence and enjoyment in PE on students’ leisure time PA through persistence. We hypothesized that both students’ perceived competence and enjoyment in PE would significantly and directly foster persistence and leisure time PA frequency. We also hypothesized that persistence would lead to more leisure time PA frequency, and function as a significant positive mediator between two exogenous variables (perceived competence and enjoyment) and PA frequency.

## Method

### Participants

This study was part of a larger research project designed to understand and enhance students’ PA involvement. Participants were 223 adolescents (115 boys, 108 girls) aged 11–15 years (*M* = 13.09, *SD* = .76 years) from four middle schools in the southwest of the U.S.A. They consisted of seventh (39.9%) and eighth (60.1%) graders. Students from the sixth grade were not included in the current study because sixth grade only accounted for 10 participants.

Upon obtaining approval from the university’s Institutional Review Board, participants provided their informed assent, while parents of all participants gave their informed consent for the students to participate. Subsequently, questionnaires were administered to participants during their regularly scheduled PE classes. Participants were told that the completion of the questionnaire was voluntary and that their identities would be kept strictly confidential. Additionally, the researchers carefully monitored participants throughout the data collection process and encouraged participants to ask questions if necessary. The questionnaires took participants approximately 25 minutes to complete.

### Measures

Participants in this study were asked to rate their level of enjoyment, perceived competence, and PA persistence using a 7-point Likert-type scale, ranging from 1 (not at all true for me) to 7 (very true for me). All of the items on the perceived competence and persistence scales began with the phrase “In my PE/athletic class…” while the items on the enjoyment scale started with “My feelings about PE and Sports …”.

#### Perceived Competence Scale

We measured perceived competence with the following four items: “I am certain I can master the skills taught in class,” “I think I am doing better than most students,” “I think I am good at PE when compared to most others,” and “I am confident I can perform as well or better than others.” The items were adapted and modified from Miller et al. (1996). We performed confirmatory factor analysis (CFA) to examine the scale’s construct validity and found an acceptable fit with the current data [Chi-square (*χ*^2^) = 3.52, *p* = .17; Chi-square degrees of freedom ratio (*χ*^2^/*df*) = 1.76; comparative fit index (CFI) = .99; Goodness-of-Fit (GFI) = .99; Standardized Root Mean Square Residual (SRMR) = .02]. The internal consistency for the scale was acceptable with a Cronbach alpha coefficient of .81.

#### Enjoyment Scale

Students responded to five items designed to assess their level of PE enjoyment. The scale was adapted and modified from Duda and Nicholls (1992), and consists of three positively worded items (“I usually enjoy playing sports and PE,” “I usually get really involved when I am playing sports and PE,” and “I usually find that time flies when I am playing sports and PE.”) and two negatively worded items (“When playing sports and PE, I am usually bored,” and “When playing sports and PE, I usually wish the game/lesson would end quickly.”). The scores from negatively worded items were reversed prior to the data analyses. The CFA revealed acceptable construct validity of enjoyment (*χ*^2^ = 27.86, *p* < .01; *χ*^2^/*df* = 5.57; CFI = .95; GFI = .95; SRMR = .04). Reliability analysis revealed that the internal consistency of enjoyment scores was good with Cronbach alpha coefficients of .85.

#### Persistence Scale

Four items (e.g., “I try to learn and do well, even if an activity is boring.”) adapted from Guan et al. (2006) were used to assess students’ self-reported persistence toward PA. The result of a CFA on this instrument yielded an acceptable fit to the current data (*χ*^2^ = 23.98, *p* < .01; *χ*^2^/*df* = 11.99; CFI = .92; GFI = .95; SRMR = .05). The Cronbach alpha coefficient for persistence scores was .80, indicating acceptable internal consistency.

#### PA Frequency Scale

To measure the direct and indirect effects of perceived competence and enjoyment on students’ leisure time PA participation through persistence as a potential mediator, we used a single-item scale adapted and modified by Godin and Shephard (1985) to measure the participants’ PA frequency during their leisure time. Participants were asked, “Considering a 7-day period (a week), during your leisure time, how often do you engage in any regular activity long enough to work up a sweat (heart beats rapidly)?” The choices were: (1) Never/Rarely, (2) Sometimes, (3) Often.

### Data Analysis

**Figure 1.**
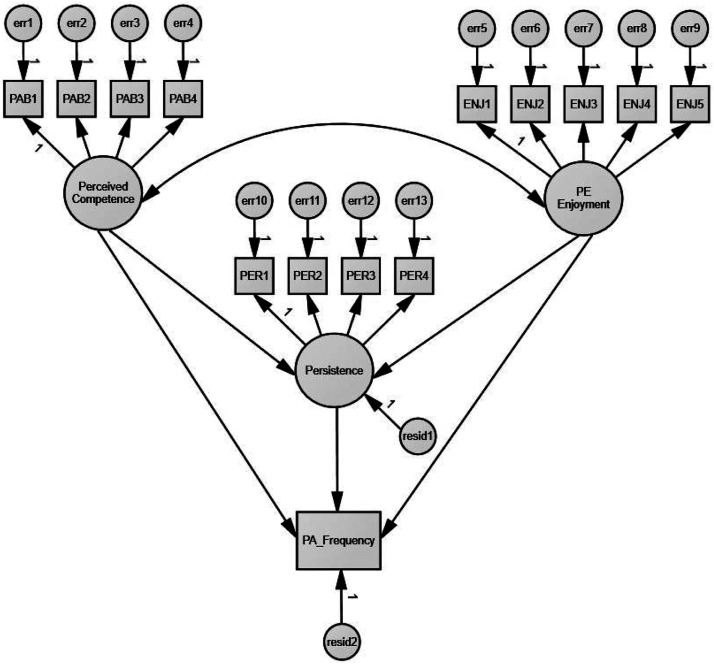
The Hypothesized Structural Model.

We calculated descriptive statistics based on gender and grade level to provide a summary of students’ perceived competence, enjoyment, persistence, and PA frequency. We employed a Pearson Product-Moment (PPM) to determine the intercorrelations between perceived competence, enjoyment, persistence, and PA frequency. We performed a 2 (Gender) x 2 (Grade level) multivariate analysis of variance (MANOVA) to examine gender and grade level differences among students’ reports of their perceived competence, enjoyment, persistence, and PA frequency. Prior to the MANOVA analysis, the assumption of homogeneity of covariance was examined using the Box’s M test. The result revealed that the observed covariance matrices of the dependent variables were equal across groups, indicating that the assumption for MANOVA was met (Box’s *M* = 19.62, *F* = .63, *p* = .94). Finally, we used a structural path model to assess the direct, indirect, and total effects of perceived competence and enjoyment on students’ leisure time PA frequency through persistence. Our hypothesized direct and indirect relationships between the exogenous variables and endogenous variables are presented in Figure 1. We employed a two-step approach recommended by Anderson and Gerbing (1988) to perform the path analysis with latent variables. In step one, a CFA was performed to develop an acceptable measurement model. In step two, once the measurement model was confirmed, the fit of the hypothesized structural path model was evaluated. A bootstrapping method with 5000 iterations was used to test the direct, indirect/mediation, and total effects. These effects were estimated using point estimates and 95% bias-corrected confidence intervals. Missing values for the perceived competence, enjoyment, and persistence were replaced with a series mean of each variable. The case with a missing value for gender, grade, and PA frequency was deleted for the purpose of accuracy in data analyses.

## Results

Table 1 presents the participants’ descriptive statistics in means and standard deviations based on the entire sample and by gender and grade level. The average participant scores for perceived competence, enjoyment, persistence, and PA frequency were all above the mid-point of their respective scales. The results of descriptive statistics also showed slightly higher scores from eighth grade students compared to seventh grade students in perceived competence, enjoyment, persistence, and PA frequency in boys, but higher scores for seventh than eighth graders among girls. Additionally, the PPM demonstrated that all four variables (perceived competence, enjoyment, persistency, & PA frequency) were significantly and positively intercorrelated (see Table 1).

**Table 1.** Descriptive Statistics and Intercorrelations Between Perceived Competence, Enjoyment, Persistence, and PA Frequency.

Variables						Male (*n* = 115)				Female (*n* = 108)			
				Full sample		Grade 7		Grade 8		Grade 7		Grade 8	
	Correlations			(*N* = 223)		(*n* = 37)		(*n* = 78)		(*n* = 52)		(*n* = 56)	
	1	2	3	*M*	*SD*	*M*	*SD*	*M*	*SD*	*M*	*SD*	*M*	*SD*
1. Competence				4.50	1.45	4.56	1.52	4.86	1.50	4.46	1.38	4.00	1.29
2. Enjoyment	.52**			5.31	1.47	5.61	1.53	5.81	1.50	4.88	1.30	4.83	1.29
3. Persistence	.63**	.52**		4.86	1.37	4.73	1.36	5.01	1.46	5.00	1.30	4.60	1.33
4. PA frequency	.44**	.42**	.39**	2.44	.59	2.35	.59	2.74	.49	2.36	.56	2.14	.55

*Note:* **p < .01.

The MANOVA showed no significant differences for Grade level (Wilk’s Lambda = .99, *F*_*(4,216)*_ = .24, *p* = .65, partial *η*^*2*^ = .01). However, the MANOVA yielded a significant interaction effect between Gender and Grade level (Wilk’s Lambda = .92, *F*_*(4,216)*_ = 4.56, *p* = .001, partial *η*^*2*^ = .08). Follow-up univariate ANOVAs only revealed a significant interaction between Gender and Grade for PA frequency (*F*_*(1,219)*_ = 16.75, *p* < .001, partial *η*^*2*^ = .07), indicating that the gender difference in PA frequency might be affected by grade level. The simple effect analysis showed that boys reported significantly higher PA frequency than girls in grade 8, (*F*_*(1,219)*_ = 40.71, *p* < .001, partial *η*^*2*^ = .15), but no significant gender difference of PA frequency was found in grade 7. Additionally, the simple effect analysis showed a significant score increase of PA frequency from grade 7 to grade 8 in boys (*F*_*(1,219)*_ = 13.17, *p* < .001, partial *η*^*2*^ = .06), but a significant score decline in girls over the same period (*F*_*(1,219)*_ = 4.55, *p* = .03, partial *η*^*2*^ = .02).

In addition to the interaction, the MANOVA yielded a significant main effect for Gender (Wilk’s Lambda = .86, *F*_*(4,216)*_ = 8.74, *p* < .001, *η*^*2*^ = .14). Follow-up univariate ANOVAs revealed that boys scored significantly higher on perceived competence (*F*_*(1,219)*_ = 5.81, *p* = .02, partial *η*^*2*^ = .03) and enjoyment (*F*_*(1,219)*_ = 19.18, *p* < .001, partial *η*^*2*^ = .08), but there was no significant gender difference for persistence (*F*_*(1,219)*_ = .14, *p* = .71, partial *η*^*2*^ = .001).

The results from CFA revealed that the original measurement model did not fit to the data (*χ*^2^ = 215.30, *p* < 0.01; *χ*^2^/*df* = 2.99; CFI = .90; GFI = .88; SRMR = .07). Examination of the modification indices suggested that the fit of the model could be improved by correlating the error terms between err6 (associated with the enjoyment variable ENJ2: “When playing sports and PE, I am usually bored”) and err8 (associated with the enjoyment variable ENJ4: “When playing sports and PE, I usually wish the game/lesson would end quickly”), and between err11 (associated with the persistence variable PER2: “Regardless of whether or not I like the activities, I work my hardest to do them”) and err13 (associated with the persistence variable PER4: “I try to learn and do well, even if an activity is boring”). After reading the test items connected to the error terms, we found that these items were either two reversed items or one similarly worded test item. According to Brown (2015), two error terms that should be correlated can be a result of reversed or similarly worded test items. After correcting the error terms, the revised measurement model provided an acceptable fit to the data (*χ*^2^ = 171.33, *p* < .01; *χ*^2^/*df* = 2.45; CFI = .93; GFI = .90; SRMR = .07).

**Figure 2.**
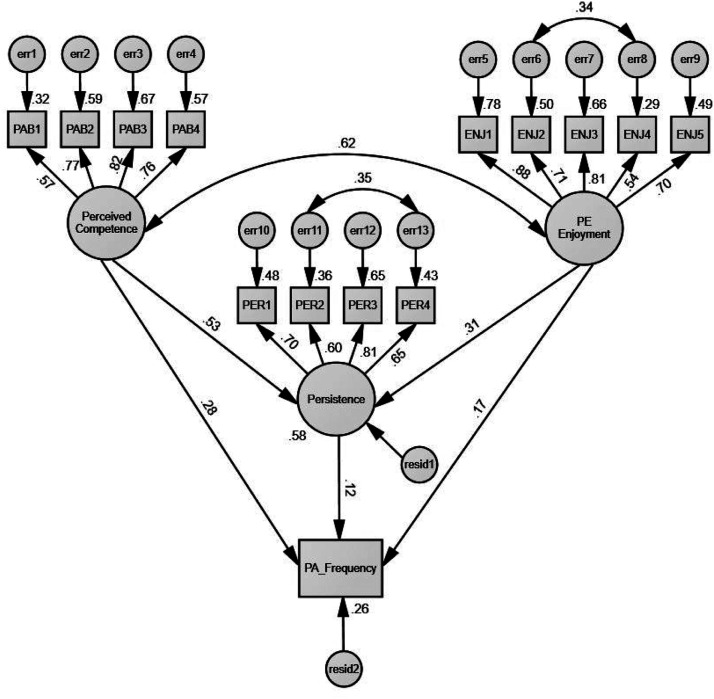
The Modified Structural Model.

The hypothesized structural path model, based on the revised measurement model, demonstrated the same good fit to the data (*χ*^2^ = 171.33, *p* < .01; *χ*^2^/*df* = 2.45; CFI = .93; GFI = .90; SRMR = .07). Figure 2 shows the path diagram and standardized path coefficients of the structural model. Specifically, 58% of the variance in persistence was accounted for by the joint influence of perceived competence and enjoyment, whereas 26% of the variance in PA frequency was explained by the combined influence of perceived competence, enjoyment, and persistence. The hypothesized structural model showed that perceived competence (*β* = .53, *t* = 4.86, *p* < .001) and enjoyment (*β* = .31, *t* = 3.42, *p* < .001) had significant direct and positive effects on persistence. Additionally, perceived competence (*β* = .28, *t* = 2.37, *p* < .02) had a significant direct and positive effect on PA frequency. However, significant direct positive effects on PA frequency were not found in enjoyment (*β* = .17, *t* = 1.83, *p* = .07) and persistence (*β* = .12, *t* = .99, *p* = .32) when controlling for the effects of perceived competence and enjoyment. Finally, both perceived competence and enjoyment mediated by persistence were related positively to PA frequency, but the indirect effects of these two exogenous variables on PA frequency were not statistically significant (see Table 2).

**Table 2.** Direct, Indirect, and Total Effects Using a Bootstrap Analysis with a 95% Confidence Interval.

Parameter	Estimate	Lower	Upper	*p*-value
Direct effect				
Competence ⇒ PA frequency	.167	.012	.306	.036
Enjoyment ⇒ PA frequency	.067	−.021	.153	.138
Competence ⇒ persistence	.693	.403	.989	.001
Enjoyment ⇒ persistence	.262	.042	.439	.022
Persistence ⇒ PA frequency	.056	−.081	.182	.376
Indirect effect				
Competence ⇒ Persistence ⇒ PA frequency	.039	−.056	.138	.347
Enjoyment ⇒ Persistence ⇒ PA frequency	.015	−.014	.067	.253
Total effect				
Competence ⇒ PA frequency	.206	.099	.319	.000
Enjoyment ⇒ PA frequency	.082	.008	.164	.032

*Note:* Unstandardized coefficients reported. Bootstrap sample = 5000 with replacement

## Discussion

We examined gender and grade differences in the relationship between students’ perceived competence, enjoyment, PA persistence, and their leisure time PA frequency. We also assessed the direct, indirect, and total effects of perceived competence and enjoyment on the students’ PA participation as mediated through persistence. In line with previous studies (Carroll & Loumidis, 2001; Ghorbani et al., 2020) and supporting our hypothesis that boys would report higher perceived competence and enjoyment in PE, our results showed that across grade levels, boys scored significantly higher on perceived competence and enjoyment in PE. These consistent findings across studies highlight the need for physical educators to be aware of gender differences in perceived competence and enjoyment in PE, as well as the important roles of these variables in enhancing middle school students’ leisure time PA participation. Furthermore, these data call for the investigation of potential factors contributing to this gender disparity. Among many factors that may account for the observed gender differences in perceived competence and enjoyment in PE, one could be gender differences in physical abilities. Given that, on average, males tend to have greater physical strength and endurance than females due to biological differences in muscle mass, it seems logical that females feel less competent and confident in PA that requires strength or endurance. Another potential factor could be gender disparities in social support. Girls may have less social support for PA than boys (Sallis et al., 2000), leading girls to feel isolated and to experience reduced PA enjoyment. However, how gender disparities in social support influence the reported gender variations in perceived competence and PA enjoyment among adolescents remains to be investigated in future research.

Contrary to previous studies (Cairney et al., 2012; Van Wersch et al., 1990) and our initial hypothesis that girls' perceptions of their competence and enjoyment in PE would decrease over time, whereas boys’ perceptions of these variables would remain stable, we did not observe significant gender differences across grade levels for either perceived competence or enjoyment. Grade differences in perceived competence and enjoyment in PE may not be fully manifested within the span of a single grade level. Moreover, our descriptive statistics suggested that eighth grade girls in our study showed lower perceived competence and enjoyment than did seventh grade girls. Based on the past findings and findings from the current study, it seems logical that girls may show a declining trend in perceived competence and enjoyment as they mature, eventually leading to significant grade-based differences, when studies have included grade 6. However, follow-up research on this topic is needed.

Partly consistent with previous research on gender differences in leisure time PA participation (Ghorbani et al., 2020), our data showed that, in grade 8, boys reported significantly higher PA frequency than girls, but there was no significant gender difference in grade 7. Three possibilities may account for these mixed findings. First, our data showed that boys had a higher perceived competence and enjoyment level in comparison to girls. According to Self-determination Theory and previous research, individuals with higher perceived competence and enjoyment increase their participation in PA. Second, eighth grade boys scored higher in perceived competence and enjoyment than seventh grade boys, but girls showed the opposite pattern. Gender-based changes with maturity may have led to the significant difference in PA frequency between girls and boys in grade 8, but not in grade 7. Lastly, girls may have relatively more social and cultural limitations to PA engagement compared to boys (Ghorbani et al., 2020). Given these mixed findings and the small number of studies available on this topic, future investigators should continue to examine gender differences in PA participation for middle school students when including grades 6 to 8.

Our study included perceived competence and enjoyment as predictors of persistence in a structural model. The results demonstrated that these two exogenous variables had a significant direct and positive effect on students’ persistence toward leisure time PA frequency. These findings confirm our hypothesis that both students’ perceived competence and enjoyment in PE would significantly and directly foster their persistence. Moreover, these results are consistent with prior research on high school students in traditional classroom settings (Hardre & Reeve, 2003; Lavigne et al., 2007), as well as with Self-Determination Theory, which suggests that that perceived competence and enjoyment can motivate people to make more effort in their assigned tasks. Additionally, in line with previous research (Cairney et al., 2012; Fairclough, 2003), our data showed that perceived competence had a significant direct and positive impact on students’ leisure time PA frequency, which supports our hypothesis that individuals with higher perceived competence are more likely to participate in PA. Given its importance and its strong link to adolescents’ PA engagement, perceived competence should be a topic for more research, especially to increase adolescents' girls’ perceived competence in PE and PA participation.

Contrary to our hypothesis, however, our data did not show that enjoyment in PE had a significant direct impact on students’ leisure time PA frequency. This was surprising since enjoyment has been identified as an important predictor of PA participation (Brazendale et al., 2015; Eberline et al., 2018) and other adaptive behaviors in sport (Puente-Díaz, 2012) and academic (Pekrun et al., 2009) settings. One reason for this finding may be our use of PA frequency as the outcome variable of interest. Brazendale et al. (2015), for example, used varied types of PA (team activities or individual activities) as outcome variables in these explorations. Perhaps types of PA are more specific to enjoyment in PE than PA frequency. Also of importance, the *p* value (*β* = .17, *t* = 1.83, *p* = .06) for the direct effects on PA frequency closely approximated statistical significance (set at *p* < .05). Moreover, the PPM correlation between enjoyment and PA frequency, as well as the total effects of enjoyment on PA frequency via the mediator of persistence *were* positively significant, indicating that enjoyment in PE is still an important motivational determinant of adolescents’ leisure time PA participation.

Although persistence was significantly and positively correlated with students’ leisure time PA frequency, we found that the indirect effects of perceived competence and enjoyment on PA frequency through persistence were not statistically significant. Furthermore, the structural model in the present study showed that persistence did not make a significant direct contribution to PA frequency when perceived competence and enjoyment were included. The results did not support our hypothesis that persistence would lead to more PA frequency and would function as a significant positive mediator. Again, a possible reason may be our outcome variable. Given that persistence may play a large role when individuals face difficulties or when strong effort is needed, it seems logical that persistence would function as a significant mediator if we used PA intensity (e.g., vigorous or moderate PA) as an outcome variable. However, PA frequency without considering PA intensity seems to be a less sensitive index. With no prior data available for persistence as a mediator variable for the motivational determinants in PE, it is premature to draw a definitive conclusion about the impact of persistence on leisure time PA engagement among middle school students. More research is needed to explore better mediators and PA outcome variables to expand the current structural path model.

### Limitations and Directions for Future Research

Several limitations should be considered when interpreting these findings. First, we relied upon students’ self-reports, limiting the objectivity of the data obtained. Second, we recognize that the sample size of each group, particularly the grade 7 boys, was relatively small, and the grade levels assessed were limited to grades 7 and 8. Therefore, generalizing the results to most middle school PE settings may be limited. Third, we used a single self-report item for PA frequency to assess PA involvement, and this may not have reflected PA intensity and duration. Future research should consider using more objective measures (e.g., heart rate monitors or accelerometers) or a complete PA scale that combines intensity, duration, and frequency. Finally, although perceived competence and enjoyment in PE are two key motivational determinants that have influences on young people’s PA participation, only 26% of the variance in PA frequency was accounted for by the joint influence of perceived competence, enjoyment, and persistence, indicating that other motivational determinants should be included in large structural equation models to predict PA behaviors. These might include, for example, achievement goals, classroom climates, and support from parents, family members, and friends.

## Conclusion

As the decline in interest and participation in PA among older adolescents and adults has become a national concern (Sallis & McKenzie, 1991), we sought to investigate motivational determinants of PA behaviors among middle school youth. We examined two key motivational determinants: perceived competence and enjoyment in PE that have been associated with middle school students’ leisure time PA behaviors. We employed a structural path model to assess the direct, indirect, and total effects of perceived competence and enjoyment on PA frequency through persistence. Our study was an expansion and addition to previous studies on the effects of motivational determinants on students’ leisure time PA participation, and our findings enhanced our understanding of PA-related motivational determinants and highlighted the need for physical educators to be aware of gender differences in perceived competence, enjoyment, and leisure time PA participation.
